# Variations in Polysomnographic Indices of Obstructive Sleep Apnea following Lingual Tonsil Hypertrophy Excision: Is the Difference Significant?

**DOI:** 10.3390/medicina58050573

**Published:** 2022-04-22

**Authors:** Ashraf Wahba, Khaled Abdelaal, Ayman Yehia, Ahmed Alsheikh, Randa Abdallah, Zakaria Ahmed, Alaa Elmazny, Mohamed Shams Eldin

**Affiliations:** 1 Department of Otorhinolaryngology, Faculty of Medicine—Damietta, Al-Azhar University, Damietta 34517, Egypt; drashrafwahba70@yahoo.com; 2 EPCRS Excellence Center, Plant Pathology and Biotechnology Lab., Faculty of Agriculture, Kafrelsheikh University, Kafrelsheikh 33516, Egypt; 3 Department of Otorhinolaryngology, Faculty of Medicine, Al-Azhar University, Cairo 11675, Egypt; aymanyabbas@yahoo.com (A.Y.); drahmed6774@gmail.com (A.A.); 4 Department of Otorhinolaryngology, Faculty of Medicine-Girls, Al-Azhar University, Cairo 11651, Egypt; randa.atwa@yahoo.com; 5 Department of Neurology, Faculty of Medicine, Al-Azhar University, Cairo 11675, Egypt; ahmedfady65@gmail.com; 6 Department of Neurology, Faculty of Medicine, Cairo University, Cairo 11956, Egypt; alaa_elmazny@kasralainy.edu.eg

**Keywords:** obstructive sleep apnea, lingual tonsil hypertrophy, polysomnography

## Abstract

*Background and Objectives*: Obstructive sleep apnea (OSA) is a sleep-related respiratory disorder that affects between 5% and 20% of the population. In obstructive sleep apnea, lingual tonsillar hypertrophy (LTH) has been suggested as a contributing factor to airway blockage. Objectives: The aim of this work is to demonstrate the polysomnographic indices and their values in OSA patients with LTH before and after the surgical intervention. *Materials and Methods*: The study was conducted on eighteen patients endoscopically diagnosed as having LTH, with the main complaints being snoring, sleep apnea, and/or sleep disturbance. Clinical examination, grading of LTH, body mass index (BMI), endoscopic assessment using Muller’s maneuver, and sleep endoscopy were recorded for all patients. The Epworth Sleepiness Scale (ESS) and overnight sleep polysomnography (PSG) were conducted before and after the surgical removal of LTH. All data were submitted for statistical analysis. *Results*: The mean ± SD of the AHI decreased from 33.89 ± 26.8 to 20.9 ± 19.14 postoperatively, and this decrease was of insignificant statistical value. The average SpO2 (%) mean ± SD was 91.14 ± 5.96, while the mean ± SD of the desaturation index was 34.64 ± 34.2. Following surgery, these indices changed to 96.5 ± 1.47 and 9.36 ± 7.58, respectively. The mean ± SD of the ESS was changed after the surgery, from 17.27 ± 6.48 to 7.16 ± 3.56. The mean ± SD of sleep efficacy was 71.2 ± 16.8 and the snoring index mean ± SD was 277.6 ± 192.37, and both improved postoperatively, to become 88.17 ± 9.1 and 62.167 ± 40.01, respectively. *Conclusions*: The AHI after lingual tonsillectomy showed no statistically significant change. The changes in the average SpO2 (%), desaturation index, sleep efficiency, snoring index, and Epworth Sleepiness Scale following the surgery were statistically significant.

## 1. Introduction

Obstructive sleep apnea (OSA) is a significant health concern, defined by recurring episodes of airflow stoppage, caused by an upper airway collapse, either total or partial. These recurring episodes of apnea and hypopnea are coupled with cyclic desaturation–reoxygenation and intermittent hypoxia, which result in the generation of reactive oxygen species, systemic vasculitis with endothelial abnormalities, sympathetic hyperactivity, and hypertension. Lingual tonsils are an aggregation of lymphoid tissue situated at the tongue base, between the circumvallate papilla and the epiglottis, alongside the glossoepiglottic fold. Hypertrophied lingual tonsils resemble a mamillated enlargement on each side of the glossoepiglottic fold. Obstructive symptoms associated with enlarged lingual tonsils often manifest as throat discomfort, recurrent throat infections, dysphonia, dysphagia, a globus feeling, snoring, and sleep apnea. In general, the frequency of LTH is low in both adult and juvenile patients with OSA. While it is possible that LTH is the etiology of obstruction in individuals with and without OSA, there is no evidence that the presence of LTH is uniquely related to OSA [1]. While adenotonsillar hypertrophy is a well-established cause of OSA in both children and adults, lingual tonsil hypertrophy is increasingly being identified as a cause of OSA, even following adenotonsillectomy (up to two-thirds of patients with LTH have undergone palatine tonsillectomy or adenoidectomy) [2]. Reactive lymphoid hyperplasia in laryngopharyngeal reflux patients, following tonsilloadenoidectomy, or in obesity, are all potential causes of LTH, but it can also be caused by medications, such as phenytoin [3]. Polysomnography is the gold standard test for the diagnosis of OSAS, and the apnea–hypopnea index (AHI) is the most frequently used index for the diagnosis and objective assessment of OSAS severity [4]. For children with OSA, lingual tonsillectomy is a successful surgical treatment that reduces the AHI by 8.9 events per hour and raises the oxygen saturation by at least 6.0% [5]. The goal of the study was to look at how the polysomnographic data changed in adults who had obstructive sleep apnea caused by lingual tonsil hypertrophy, following its surgical resection.

## 2. Materials and Methods

**Figure 1:**
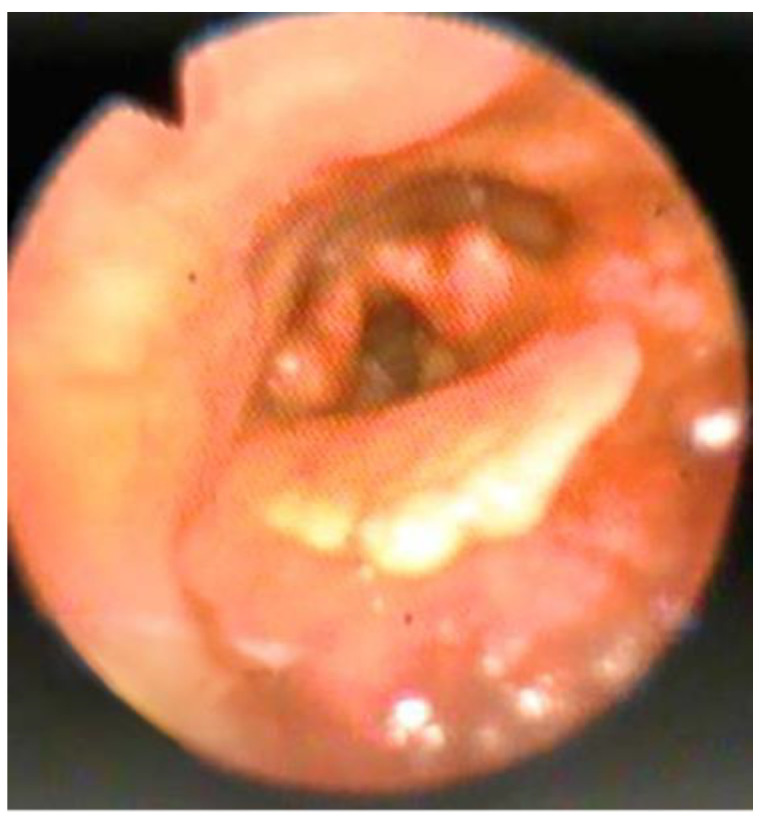
Nasopharyngosopic view of patient with grade 4 LTH.

This research was conducted at the Al-Ansari hospital in Yanbu, Saudi Arabia, between 2019 and 2021. Following obtaining their informed consent, eighteen subjects were recruited for this study. Patients with significant nasal septal deviation, nasal mass, or nasal polyp, as well as those with central sleep apnea, congenital facial deformity, a neuromuscular condition, or a documented degree of obstruction other than in the retro-lingual region, were excluded from the study. Each patient who complained of snoring, sleep apnea, or sleep disruption was subjected to a thorough analysis of their medical history, a clinical examination, and a body mass index assessment (BMI). It was calculated using the following formula: BMI = $\frac{\mathrm{Weight}}{{\mathrm{Hight}}^{2}}$. Patients were categorized according to their BMI in the following manner: underweight ranges from 16.0 to 18.5, normal ranges from 18.5 to 25, overweight ranges from 25 to 30, moderately obese ranges from 30 to 35, severely obese ranges from 35 to 40, and very severely obese ranges from 35 to 40. The Epworth Sleepiness Scale (ESS) was used to assess daytime drowsiness [6]. Upper airway endoscopy was conducted in the supine posture to diagnose the location of OSA. To confirm retro-lingual collapse, an endoscopic examination, utilizing a flexible fiberoptic endoscope and Muller’s maneuver, was performed. While the patient was upright, with a closed mouth and a pinched nose, Muller’s procedure was conducted with maximal inspiration. The lingual tonsillar tissue was graded based on the Friedman LTH grading system. The grading system consists of a scale from 0 to 4. Grade 0 is defined as having no lymphoid tissue. Dispersed tongue-based lymphoid tissue is classified as Grade 1. Grade 2 is lymphoid tissue covering the entire base of the tongue. Grade 3 is lymphoid tissue with a thickness of 5–10 mm, covering the tongue base. Grade 4 (Figure 1) is lymphoid tissue that is at least 1 cm thick, raising the epiglottis [7]. Surgical excision of the lingual tonsil under general anesthesia, with nasotracheal intubation by combined cold knife dissection and bipolar coagulation diathermy, was performed on the patients with grade 3 or grade 4 LTH. PSG was performed on all the participants in the Sleep Lab Unit of the Al-Ansari Specialized Hospital (Xltek^®^ Brain Monitor with Natus^®^ SleepWorks™ PSG software) overnight. Following the onset of sleep, participants were connected to a set of electrodes that included electroencephalography, electrocardiography, and electrooculography, as well as chin electromyogram (EMG), leg EMG, respiratory flow and effort sensors, and an oxygen saturation sensor. The collected data were recorded and processed by the computer after the participants fell asleep. During sleep, apnea (respiratory airflow collapse) and hypopnea (respiratory airflow reduction) were recognized and measured. The scoring was carried out in accordance with the American Association of Sleep Medicine’s 2020 scoring criteria. Based on the apnea–hypopnea index values, patients were divided into those with no sleep apnea (AHI < 5), those with mild sleep apnea (AHI 5–15), those with moderate sleep apnea (15–30), and those with severe sleep apnea (AHI > 30) [8]. Overnight sleep studies were undertaken prior to, and six months after, surgical excision of the lingual tonsils. PSG results, apnea–hypopnea index (AHI), average SpO2 (%), desaturation index, sleep efficacy, and snoring index were compared preoperatively and postoperatively. The Epworth Sleepiness Scale questionnaire was filled out during the follow-up session. Statistical analysis of the data: Statistics were conducted using a computer with SPSS data editor software for Windows, version 25.0 (IBM SPSS Statistics, IBM Corp., Armonk, NY, USA). The tests used were X mean and standard deviation (SD) to measure the central tendency of data and the distribution of data around the mean. Student’s *t*-test was used to identify if there was a statistically significant difference between the means of two samples. When *p* > 0.05, the data were considered significant.

## 3. Results

**Figure 2:**
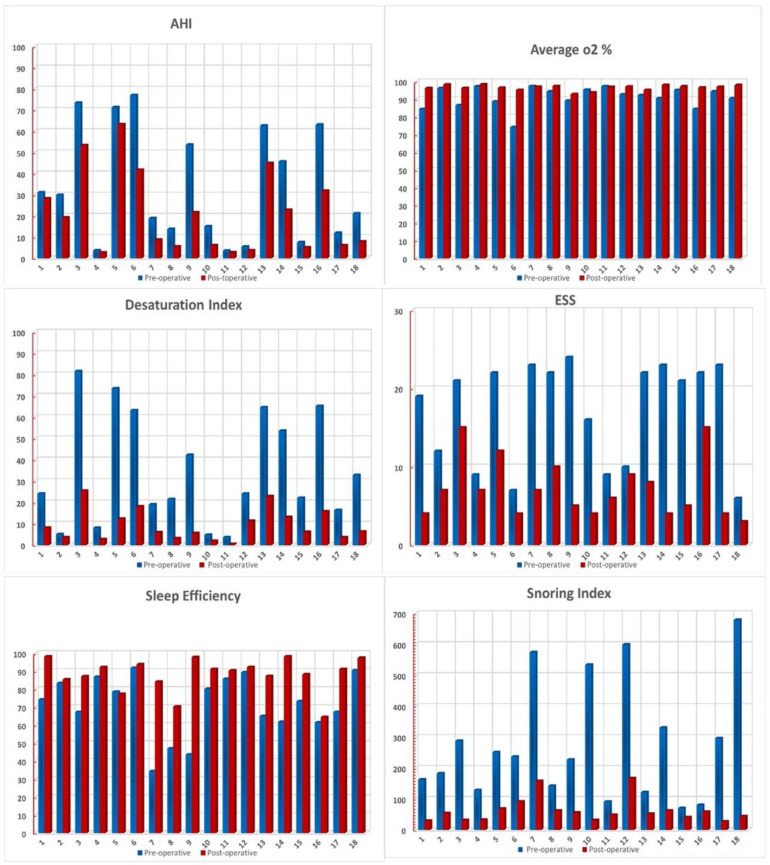
Graphic presentation of the pre- and postoperative values of the studied cases.

**Figure 3:**
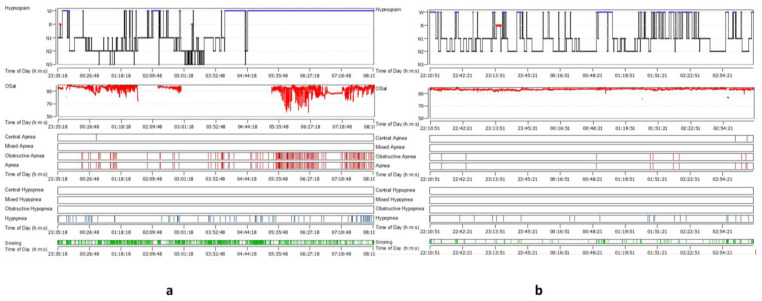
(**a**) PSG of patient with severe OSA, LTH grade 4 before the operation; (**b**) PSG after lingual tonsillectomy.

In this study, a total of 18 individuals (16 males and 2 females) were enrolled, all of whom were found to have an LTH after an office-based endoscopic examination. For this study, only patients with LTH gradings of 3 and 4 were included. Patients with grade 4 LTH (Figure 1) were diagnosed in 13 cases (72.3%), whereas grade 3 LTH was detected in 5 cases (27.7%). The mean age was 42 years old, with a range of 28 to 58 years across the participants. BMI was distributed as follows: three overweight (16%), five moderately obese (27.5%), five severely obese (27.5%), and five very severely obese (27.8%). The mean ± SD of the BMI (kg/m^2^) was 38.64 ± 12.48. Polysomnographic data were collected preoperatively and showed that three patients (16.6%) had mild obstructive sleep apnea, six (33.3%) had moderate obstructive sleep apnea, and seven (38.9%) had severe obstructive sleep apnea, according to the AHI. Two patients (11.1%) with complaints of snoring and sleep difficulties linked to LTH had an AHI of less than five. The mean ± SDAHI was 33.89 ± 26.8. The average SpO2 (%) mean ± SD was 91.14 ± 5.96, whereas the mean ± SD of the desaturation index was 34.64 ± 34.2. The mean ± SD of sleep efficacy was 71.2 ± 16.8, while the snoring index mean ± SD was 277.6 ± 192.37. The Epworth Sleepiness Scale questionnaire was reported for all cases in the study at the time of diagnosis and in the follow-up. Five patients (27.7%) were found to be not sleepy, one patient (5.5%) was considered mildly sleepy, and twelve patients (66.7%) were very sleepy. The mean ± SD of the ESS was 17.27 ± 6.48. The polysomnographic indices following lingual tonsillectomy showed a decrease in the mean ± SD of AHI to 20.9 ± 19.14. Six patients (33.3%) had mild OSA, four patients (22.2%) had moderate OSA, and five patients had severe OSA (27.7%). In three cases, the AHI was less than five (16.6%). The change in the mean AHI did not differ statistically from the baseline (*p* value 0.175). The mean ± SD of the average SpO2 (%) and the snoring index postoperatively were increased to 96.5 ± 1.47 and 62.16 ± 40.08, respectively, while that of the desaturation index was decreased to 9.36 ± 7.58. These variations between the preoperative and postoperative readings were statistically significant. The sleep efficiency and the ESS mean ± SD were changed to 88.17 ± 9.1 and 7.16 ± 3.56, respectively, from their previous values. The difference between the pre- and postoperative mean of sleep efficiency and the Epworth Sleepiness Scale was statistically significant (*p* value 0.015 and 0.018, respectively) (Figure 2 and Figure 3 and Table 1).

## 4. Discussion

The relationship between LTH and OSA in adults has not been frequently investigated in the literature. The association is not well evident in all studies, and the link between them is doubtful. Polysomnographic indices, mainly the AHI, are a reliable measure to assess the diagnosis of OSA, and to study the relation between LTH grading and OSA. In an attempt to study the changes in the PSG parameters following lingual tonsil hypertrophy surgery in adult OSA patients with grade 3 and 4 LTH, we evaluated the PSG measures and the Epworth Sleepiness Scale before and after lingual tonsillectomy for 18 OSA patients who had an office-based endoscopic confirmed diagnosis of LTH. In the current study, the mean age of the patients was 42.5 ± 9.58 and the BMI kg/m^2^ mean ± SD was 38.64 ± 12.48. Males accounted for 88.8% of the studied cases of LTH, while only two females (10.2%) were observed in the study. It is worth noting that this study had a disproportionately male sample. As a result, the differences between the gender-balanced subgroups are difficult to discern. We also found a positive relation between OSA severity, LTH grading, and BMI. Age-related resistant fat deposition, palatal and oropharyngeal redundancy associated with a high BMI, and the larger number of males may have contributed to this positive relation. Of the studied cases, 72.3% were diagnosed as grade 4 LTH, and 27.7% of the cases were diagnosed as grade 3 LTH. These frequencies were also observed in a study on the significance of LTH and OSA severity, where the incidence of LTH in 644 adult patients suffering from OSA was found to be 5.6% (36 patients). The mean ± SD of their age and BMI kg/m^2^ was 50.66 ± 9.75 and 33.21 ± 5.62, respectively. They determined the mean ± SD of the age and BMI kg/m^2^ of OSA patients without LTH to be 54.41 ± 10.60 and 32.80 ± 5.03, respectively [9]. They concluded that, despite the estimation of a significantly smaller retroglossal airway, with evident radiological proof of reduced airway dimensions in OSA patients with LTH, compared to OSA patients without LTH, it did not affect the severity of OSA (the mean AHI in apneic patients without LTH was 43.24, and in apneic patients with LTH, it was 40.62). According to the results of the present study, we can confirm the significant correlation between lingual tonsillectomy and the improvement in various oxygen saturation indices, despite the insignificant reduction in the AHI after surgery. The mean ± SD of the AHI in the current study, on patients with complaints of snoring and sleep disorders associated with LTH, was 33.89 ± 26.8. This was changed to 20.9 ± 19.14 after the surgery, with persistent severe OSA in 27.7% of patients. However, this change was not statistically significant. These findings suggest that LTH may reduce the retroglossal airway, but with no considerable impact on the AHI. Tang and Friedman [1] also concluded, in a retrospective study of 93 LTH patients, that LTH is uncommon, even in those with OSA, and does not differ between patients with and without OSA [1]. In our study, we found a statistically significant difference in the patients’ preoperative and postoperative values of average SpO2 (%) and desaturation index (DI). The mean ± SD of the average SpO2 (%) was increased postoperatively from 91.14 ± 5.96 to 96.5 ± 1.47, while that of the desaturation index was decreased from 34.64 ± 34.2 to 9.36 ± 7.58. The mean average oxygen desaturation was increased by 5.5% after the surgery. Lee et al. [10] and Li et al. [11] observed the same findings, where minimal oxygen saturation showed a statistically significant difference between the preoperative and postoperative values. Similar findings were found in a meta-analysis of the results of four studies on pediatric sleep apnea in relation to LTH [12]. The mean change in the AHI after lingual tonsillectomy was a reduction of 8.9 events per hour. The mean improvement in the minimal oxygen desaturation level, following lingual tonsillectomy, was 6.0%. Overall, 17% of the patients had a postoperative AHI of less than one, and 51% had a postoperative AHI of less than five. The sleep efficiency and ESS mean ± SD were changed from 71.2 ± 16.8 and 17.27 ± 6.48 to 88.17 ± 9.1 and 7.16 ± 3.56, respectively, and the difference between these means, following the surgery, was statistically insignificant. Similarly, Lee et al. [10] and Verse et al. [13] identified ESS as a clinical estimate for the efficiency of sleepiness in two trials (74 patients). In addition, a review of the data, following lingual tonsillectomy with palatal surgery, revealed a statistically significant improvement in the ESS, with an average reduction of 5.44 [14]. The results of the current study have been supported by a recent study, which showed a significant statistical decrease in the ESS score from 11 ± 5.11 to 7.9 ± 4.94 (*p* < 0.05) after ablation of the lingual tonsils using coblation technology, as part of multilevel surgery performed in a study on 24 patients. This improvement was associated with a significant reduction in the mean AHI from 33.01 ± 17 to 17.7 ± 13 postoperatively (*p* < 0.05) [15]. The difference between the preoperative and postoperative mean ± SD of the snoring index was statistically significant. The mean ± SD of the snoring index was 277.6 ± 192.37, following the surgery, to 62.16 ± 40.08. Correlated changes in these indices have been associated with the severity of obstructive sleep apnea. A direct relationship between the severity of obstructive sleep apnea and the AHI and SpO2 parameters, including the desaturation index, was observed in a retrospective study that used data from an obstructive sleep apnea survey that was conducted among the middle-aged Saudi population, which may suggest their potential as predictors of the severity of obstructive sleep apnea [16]. Different surgical modalities were reported to be used for the resection of lingual tonsil hypertrophy, including cauterization, surgical cold knife, LASER, coblation tongue base reduction, and cryosurgery, with variable degrees of intraoperative and postoperative complications, including bleeding, postoperative infection, pain, and compromised airways. Except for prolonged pain and accepted intraoperative bleeding that can be controlled with bipolar cautery, we do not report any major complications, such as airway embarrassment or wound infection. Similarly, Mure et al. [16] reported no major operative or postoperative complications after surgical excision of LTH using a diode laser or coblation. Due to the study’s small sample size and retrospective design, as well as the fact that all the study participants were selected primarily on the basis of clinical findings, the authors recommend the following prospective design to be used in future studies: a larger sample size; case selection based on the characteristic of obstructive evidence from drug-induced sleep endoscopy, confirming the diagnosis of LTH as an obstructing cause; and long-term evaluation of the quality of life following various surgical modalities of treatment of LTH associated with OSA.

## 5. Conclusions

Following lingual tonsillectomy, adult OSA patients, when observed, showed no statistically significant changes in relation to the AHI. However, the difference between the average SpO2 (%), desaturation index, sleep efficiency, snoring index, and Epworth Sleepiness Scale was statistically significant. Lingual tonsillectomy could be an effective procedure in cases of grade 4 hypertrophy, and the improvement in polysomnographic indices (other than AIH), together with the sleepiness scale, was remarkable.

## Figures and Tables

**Table 1:** Statistical analysis between preoperative and postoperative polysomnographic indices among LTH patients.

	Pre-Operative	Post-Operative	*p*-Value	*t*-Test
AHI	33.89 ± 26.8	20.9 ± 19.14	0.1754	1.9604
Average o2	91.14 ± 5.96	96.51 ± 1.47	0.001	16.34
Desaturation index	34.61 ± 34.22	9.36 ± 7.58	0.0002	20.354
Sleep Efficiency	71.21 ± 16.84	88.17 ± 9.1	0.0152	3.4239
Snoring Index	277.6 ± 192.37	62.16 ± 40.08	0.0001	23.111
Epworth	17.44 ± 6.48	7.16 ± 3.56	0.0181	3.3054
Age	42.5 ± 9.5			
BMI	38.64 ± 2.5			

## Data Availability

The data presented in this study are available on request from the corresponding author. The data are not publicly available due to terms of privacy.
